# Supplementary material for: HIVprotI: an integrated web based platform for prediction and design of HIV proteins inhibitors
Source: J Cheminform. 2018 Mar 9;10:12. doi: 10.1186/s13321-018-0266-y (PMC5845081; doi:10.1186/s13321-018-0266-y)
Supplement: Supplementary file 2 — Additional file 2. Source code of HIVProtI web server. [file 13321_2018_266_MOESM2_ESM.zip › HIVprotI_Source-code/hivprotI/weka-3-6-6/doc/weka/core/TechnicalInformation.html]

TechnicalInformation


---


|  |  |  |
| --- | --- | --- |
| **Overview** \| **Package** \| **Class** \| **Tree** \| **Deprecated** \| **Index** \| **Help** \| **Weka's home** | |  |
| **PREV CLASS**   **NEXT CLASS** | **FRAMES**    **NO FRAMES**     **All Classes** |  |
| SUMMARY: NESTED \| FIELD \| CONSTR \| METHOD | DETAIL: FIELD \| CONSTR \| METHOD |  |


---


## weka.core Class TechnicalInformation

```
java.lang.Object
  weka.core.TechnicalInformation
```

**All Implemented Interfaces:**: RevisionHandler

---

``` public class TechnicalInformation extends java.lang.Object implements RevisionHandler ```

Used for paper references in the Javadoc and for BibTex generation.
Based on documentation found here:

http://www.ecst.csuchico.edu/~jacobsd/bib/formats/bibtex.html

BibTex examples can be found here:

http://bib2web.djvuzone.org/bibtex.html

**Version:**
:   $Revision: 1.11 $

**Author:**
:   fracpete (fracpete at waikato dot ac dot nz)

**See Also:**: `TechnicalInformationHandler`

---

| **Nested Class Summary** | |
| --- | --- |
| `static class` | `TechnicalInformation.Field`             the possible fields |
| `static class` | `TechnicalInformation.Type`             the different types of information |


**Constructor Summary**

`TechnicalInformation(TechnicalInformation.Type type)`
  
          Initializes the information with the given type

`TechnicalInformation(TechnicalInformation.Type type,
java.lang.String id)`
  
          Initializes the information with the given type


| **Method Summary** | |
| --- | --- |
| `TechnicalInformation` | `add(TechnicalInformation.Type type)`             Adds an empty technical information with the given type to the list of additional informations and returns the instance. |
| `void` | `add(TechnicalInformation value)`             adds the given information to the list of additional technical informations |
| `java.util.Enumeration` | `additional()`             returns an enumeration of all the additional technical informations (if there are any) |
| `boolean` | `exists(TechnicalInformation.Field field)`             returns TRUE if the field is stored and has a value different from the empty string. |
| `java.util.Enumeration` | `fields()`             returns an enumeration over all the stored fields |
| `java.lang.String` | `getID()`             returns the unique ID (either the one used in creating this instance or the automatically generated one) |
| `java.lang.String` | `getRevision()`             Returns the revision string. |
| `TechnicalInformation.Type` | `getType()`             returns the type of this technical information |
| `java.lang.String` | `getValue(TechnicalInformation.Field field)`             returns the value associated with the given field, or empty if field is not currently stored. |
| `boolean` | `hasAdditional()`             returns true if there are further technical informations stored in this |
| `static void` | `main(java.lang.String[] args)`             Prints some examples of technical informations if there are no commandline options given. |
| `void` | `setValue(TechnicalInformation.Field field, java.lang.String value)`             sets the value for the given field, overwrites any previously existing one. |
| `java.lang.String` | `toBibTex()`             Returns a BibTex string representing this technical information. |
| `java.lang.String` | `toString()`             Returns a plain-text string representing this technical information. |

**Methods inherited from class java.lang.Object**

`equals, getClass, hashCode, notify, notifyAll, wait, wait, wait`

**Constructor Detail**

### TechnicalInformation

```
public TechnicalInformation(TechnicalInformation.Type type)
```

:   Initializes the information with the given type

    **Parameters:**: `type` - the type of this information **See Also:**: `TechnicalInformation.Type`

---


### TechnicalInformation

```
public TechnicalInformation(TechnicalInformation.Type type,
                            java.lang.String id)
```

:   Initializes the information with the given type

    **Parameters:**: `type` - the type of this information: `id` - the unique ID (for BibTex), can be empty **See Also:**: `TechnicalInformation.Type`


**Method Detail**

### getType

```
public TechnicalInformation.Type getType()
```

:   returns the type of this technical information

    :   **Returns:**: the type of this information

---


### getID

```
public java.lang.String getID()
```

:   returns the unique ID (either the one used in creating this instance
    or the automatically generated one)

    :   **Returns:**: the ID for this information

---


### setValue

```
public void setValue(TechnicalInformation.Field field,
                     java.lang.String value)
```

:   sets the value for the given field, overwrites any previously existing one.

    :   **Parameters:**: `field` - the field to set the value for: `value` - the value of the field

---


### getValue

```
public java.lang.String getValue(TechnicalInformation.Field field)
```

:   returns the value associated with the given field, or empty if field is
    not currently stored.

    :   **Parameters:**: `field` - the field to retrieve the value for **Returns:**: the value associated with this field, empty if not existing

---


### exists

```
public boolean exists(TechnicalInformation.Field field)
```

:   returns TRUE if the field is stored and has a value different from the
    empty string.

    :   **Parameters:**: `field` - the field to check **Returns:**: true if a value is stored for the field and non-empty

---


### fields

```
public java.util.Enumeration fields()
```

:   returns an enumeration over all the stored fields

    :   **Returns:**: all currently stored fields

---


### hasAdditional

```
public boolean hasAdditional()
```

:   returns true if there are further technical informations stored in this

    :   **Returns:**: true if there are further technical informations available

---


### additional

```
public java.util.Enumeration additional()
```

:   returns an enumeration of all the additional technical informations (if
    there are any)

    :   **Returns:**: an enumeration over all additional technical informations

---


### add

```
public void add(TechnicalInformation value)
```

:   adds the given information to the list of additional technical
    informations

    :   **Parameters:**: `value` - the information to add

---


### add

```
public TechnicalInformation add(TechnicalInformation.Type type)
```

:   Adds an empty technical information with the given type to the list
    of additional informations and returns the instance.

    :   **Parameters:**: `type` - the type of the new information to add **Returns:**: the generated information

---


### toString

```
public java.lang.String toString()
```

:   Returns a plain-text string representing this technical information.
    Note: it only returns a string based on some fields. At least AUTHOR,
    YEAR and TITLE are necessary.

    :   **Overrides:**: `toString` in class `java.lang.Object`
    :   **Returns:**: a string representation of this information

---


### toBibTex

```
public java.lang.String toBibTex()
```

:   Returns a BibTex string representing this technical information.
    Note: this is just a very raw implementation, special characters need to
    be escaped manually for LaTeX.

    :   **Returns:**: the BibTeX representation of this information

---


### getRevision

```
public java.lang.String getRevision()
```

:   Returns the revision string.

    :   **Specified by:**: `getRevision` in interface `RevisionHandler`
    :   **Returns:**: the revision

---


### main

```
public static void main(java.lang.String[] args)
                 throws java.lang.Exception
```

:   Prints some examples of technical informations if there are no
    commandline options given. Otherwise the information of a given
    TechnicalInformationHandler can be printed.

    Valid options are:

    -W classname


---


|  |  |  |
| --- | --- | --- |
| **Overview** \| **Package** \| **Class** \| **Tree** \| **Deprecated** \| **Index** \| **Help** \| **Weka's home** | |  |
| **PREV CLASS**   **NEXT CLASS** | **FRAMES**    **NO FRAMES**     **All Classes** |  |
| SUMMARY: NESTED \| FIELD \| CONSTR \| METHOD | DETAIL: FIELD \| CONSTR \| METHOD |  |


---
